# Low Vitamin D in Narcolepsy with Cataplexy

**DOI:** 10.1371/journal.pone.0020433

**Published:** 2011-05-25

**Authors:** Bertrand Carlander, Anne Marie Puech-Cathala, Isabelle Jaussent, Sabine Scholz, Sophie Bayard, Valérie Cochen, Yves Dauvilliers

**Affiliations:** 1 National Reference Network for Narcolepsy, Department of Neurology, Hôpital Gui-de-Chauliac, CHU Montpellier, Montpellier, France; 2 Laboratoire d'Hormonologie, Hôpital Lapeyronie, CHU Montpellier, Montpellier, France; 3 Inserm U1061, University of Montpellier 1, Montpellier, France; Chiba University Center for Forensic Mental Health, Japan

## Abstract

**Background:**

Narcolepsy with cataplexy (NC) is currently thought to be an autoimmune-mediated disorder in which environmental risk factors make a significant contribution to its development. It was proposed that vitamin D deficiency plays a role in autoimmune diseases. Here we investigated whether NC can be associated with 25-hydroxyvitamin D (25(OH)D) level deficiency in patients with NC compared with gender- and age-matched normal controls.

**Methodology:**

Serum level of 25 (OH)D was determined in 51 European patients with typical NC compared to 55 age-, gender-, and ethnicity-matched healthy controls. Demographic and clinical data (age at onset, duration and severity of disease at baseline, and treatment intake at time of study) and season of blood sampling were collected to control for confounding variables.

**Principal Findings:**

Serum 25(OH)D concentration was lower in NC compared to controls (median, 59.45 nmol/l [extreme values 24.05–124.03] vs. 74.73 nmol/l [26.88–167.48] p = 0.0039). Patients with NC had significantly greater vitamin D deficiency (<75 nmol/l) than controls (72.5% vs 50.9%, p = 0.0238). Division into quartiles of the whole sample revealed that the risk of being affected with NC increased with lower 25(OH)D level, with a 5.34 OR [1.65–17.27] for the lowest quartile (p = 0.0051). Further adjustment for BMI did not modify the strength of the association (OR: 3.63, 95% CI = 1.06–12.46, p = 0.0191). No between BMI and 25(OH)D interaction, and no correlation between 25(OH)D level and disease duration or severity or treatment intake were found in NC.

**Conclusion:**

We found a higher frequency of vitamin D deficiency in NC. Further studies are needed to assess the contribution of hypovitaminosis D to the risk of developing narcolepsy, and to focus on the utility of assessing vitamin D status to correct potential deficiency.

## Introduction

Narcolepsy with cataplexy (NC), a disease caused by loss of hypocretin cells in the hypothalamus, is currently thought to be an autoimmune-mediated disorder [1]. This hypothesis is based on the close association between the HLA-DRB1*1501-DQB1*0602 haplotype [2], the strongly protective DRB1*1301-DQB1*0603 haplotype [3], the finding of polymorphisms in the T-cell receptor alpha locus [2], [3], peripubertal age at onset [4], the presence of elevated Tribbles homolog 2 antibody levels [5], and the positive effect of intravenous IgG to normalize CSF hypocretin-1 level reported in a single patient with NC [6]. Thus, loss of hypocretin-containing neurons might be attributable to an autoimmune process. Although the precise etiology of NC remains unknown, it is clear that both genetic and environmental factors are important [1], [7].

Vitamin D has been increasingly recognized for its pleiotropic actions [8]. Vitamin D deficiency has been implicated in a range of skeletal and cardiovascular diseases as well as numerous autoimmune diseases [8]. Vitamin D receptors are present in the brain (microglia) and in circulating immunity cells, and recent studies confirmed this vitamin's role in autoimmunity and neuroprotection [8], [9]. Vitamin D has been proposed as a key environmental factor for autoimmune diseases, especially in multiple sclerosis (MS) [9], another disease strongly associated with the HLA-DRB1*1501-DQB1*0602 haplotype. Little is known about the precise environmental triggers in NC.

The aim of this study was to investigate 25-hydroxyvitamin D (25(OH)D) level in European patients with narcolepsy with typical cataplexy compared with gender- and age-matched normal controls.

## Materials and Methods

### Subjects

All subjects gave their written informed consent to participate in the study, which was approved by the Montpellier University Hospital's ethics committee.

Fifty-one European patients with NC (22 males and 29 females, 16–65 y.o., median age 34.0) were included in the study. Diagnosis was according to the revised International Classification of Sleep Disorders (ICSD-2) [10]. All patients underwent one night of polysomnography recording [11] followed by multiple sleep latency tests (MSLT) the next day, consisting of five naps scheduled at two-hour intervals starting at 9:00 a.m [12]. NC was diagnosed based on the presence of excessive daytime sleepiness, clear-cut and frequent cataplexy, HLA DQB1*0602 positivity, and at least two sleep onset REM periods (SOREMPs) during the MSLT [12]. No patients had any psychiatric disorder based on the DSM-IV criteria or any other significant comorbid condition

We recruited 55 normal European subjects matched for age and gender (20 males and 35 females, 21–62 y.o., median age 36.0). All healthy controls were community-dwelling adults living in the same area in Southern France. Inclusion criteria for controls were the ability to understand and give informed consent, no history of psychiatric or neurological or other organic disease, and no daytime sleepiness (Epworth Sleepiness Scale <11).

Demographic data (age, gender, and body mass index-BMI) and season of blood sampling were collected on patients and controls. Obesity was assessed on the basis of BMI calculated as weight/height2 (kg/m2). Subjects were classified as normal, overweight or obese according to body mass index <25, ≥25, respectively [13]. Clinical data (age at onset 6–48 years, duration of disease 0–49 years, Epworth Sleepiness Scale, and frequency of cataplectic attacks), polysomnographical data (mean sleep latency and number of SOREMPs on the MSLT) at baseline, and the presence of treatment intake at time of study were gathered on the patients to control for known and potential confounding variables. Medication at the time of study was noted in all patients: 33 patients were untreated and 18 were taking stimulants and/or anticataplectic drugs.

### Procedures

#### Blood and CSF sampling

Patients and controls underwent fasting for venous blood sampling between 7:00 and 8:00 a.m. following standardized procedures at the Montpellier Sleep Disorders Center. Sera were separated and frozen immediately for further analysis and were handled similarly between patients and controls before assay. A lumbar puncture was performed in 19 patients with narcolepsy-cataplexy to measure CSF hypocretin-1 level. Hypocretin-1 peptide level was determined in duplicate from CSF samples without prior extraction using ^125^I radioimmunoassay kits (Phoenix Pharmaceuticals, Inc., Belmont, CA) according to manufacturer's instructions. Low CSF hypocretin-1 level, i.e. <110 pg/ml [1], was found in all patients.

Concentration of 25-hydroxyvitamin D ergocalciferol (D2) and cholecalciferol (D3) (25(OH)D) was measured by radioimmunoassay (25OH Vitamin D RIA, IDS, Immunodiagnostic Systems, Boldon, UK). Interassay coefficient of variation was <8.2% and intraassay coefficient was <6.1%. Samples were run as single assays. The commonly accepted international norm for 25(OH)D serum level is >75 nmol/l [9], [14].

### Statistical methods

The sample is described using percentages for categorical variables and median and range for quantitative variables. Distributions were skewed according to the Shapiro-Wilk test. Chi square tests were used to compare categorical variables between the two groups and the Mann-Whitney test was used for continuous variables. Spearman's rank order correlations were applied to measure associations between two continuous variables. Univariate comparisons between controls and patients with NC were made using logistic regression analysis and quantified with (OR) and their 95% confidence intervals (CI). Continuous variables 25(OH)D were divided into sample quartiles and also taken with a clinical cut-off of 75 nmol/l. Multivariate logistic regression analyses were performed on clinical and social variables with p-values <0.1 in the univariate analysis. When appropriate, interaction terms were tested using the Wald χ^2^ test statistic given by the logistic regression model. Significance level was set at p<0.05. Statistical analyses were performed using SAS software, version 9.2 (SAS Institute, Cary, NC, USA).

## Results

Variable results on demographic, clinical, and season of blood sampling are summarized in Table 1. No group difference was observed except for higher BMI in patients with NC (p<0.0022), results expected as controls came from the general population. Hence, 27.5% of patients were obese (BMI >30 kg/m^2^) in contrast to 5.5% of controls. There was neither significant association between gender (median 25(OH)D level (men, 64 [extreme values 28.38–123.40], and women, 71.43 [24.05–167.48], p = 0.42) 25(OH)D level in the whole population nor age effect (median 25(OH)D level (Age<26 y.o., 64.78 [28.38–167.48], Age 26–34, 61.70 [26.88–116.78], Age 35–48, 84.58 [24.05–124.03], and Age > 48, 64.13 [31.40–154.43], p = 0.194). However a significant correlation was found between BMI and 25(OH)D (r = −0,40, p<0.0001).

**Table 1:** Description of socio-demographic and clinical variables between normal controls and patients with narcolepsy-cataplexy.

	Normal controls N = 55		Narcolepsy with cataplexy N = 51		
	n	%	n	%	p-value
Gender					
Male	20	36.36	22	43.14	0.48
Female	35	63.64	29	56.86	
Age (years)					
<26	11	20.00	13	25.49	0.92
[26–35[	15	27.27	13	25.49	
[35–49[	14	25.45	13	25.49	
≥49	15	27.27	12	23.53	
BMI (kg/m^2^)					
<25	43	78.18	25	49.02	0.0022
≥25	12	21.82	26	50.98	
Season of sampling					
Winter	12	21.82	12	23.53	0.13
Spring	17	30.91	15	29.41	
Summer	22	40.00	13	25.49	
Fall	4	7.27	11	21.57	

BMI: Body Mass Index.

Between-season sampling differences were also seen for the median 25(OH)D level (Winter, 41.53 [extreme values 24.05–110.95], Spring, 69.28 [34.00–167.48], Summer, 82.40 [33.95–154.43], and Fall, 57.18 [33.90–123.83], p<0.0001).

**Figure 1:**
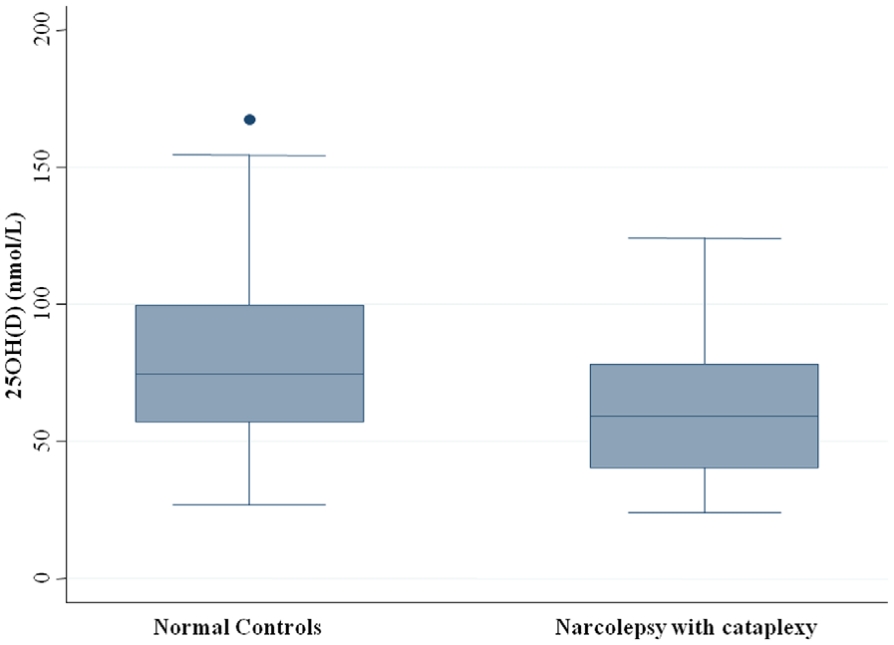
Sera measurement of 25-hydroxyvitamin D (25(OH)D) level (in nmol/L) in patients with narcolepsy-cataplexy and age- and gender-matched normal controls. Results are shown as a box-whisker plot, with median and 25th quartile of 25(OH)D. We noted significantly lower level in patients compared to controls (P = 0.0039).

Figure 1 illustrates the distribution of sera 25(OH)D level in patients with NC and in controls. A significantly lower level is seen in patients compared to normal controls (median, 59.45 nmol/l [24.05–124.03] vs. 74.73 nmol/l [26.88–167.48] p = 0.0039). Vitamin D deficiency, defined as <75 nmol/l, was found in 72.5% of patients compared to 50.9% of controls (p = 0.0238; OR: 2.55, 95% CI = 1.13–5.73). Moreover, dividing the whole population into quartiles revealed that the risk of being affected with narcolepsy-cataplexy increased with lower 25(OH)D level, with a 5.34 odds ratio [1.65–17.27] for the lowest quartile (p = 0.0051) (Table 2). A low vitamin D dose-response relationship was also found in patients with NC (p trend = 0.0023). Further adjustment for BMI (classified on two classes <25 and ≥25 kg/m^2^) did not modify the strength of the association (p trend = 0.0191; odds ratio: 3.63, 95% CI = 1.06–12.46) (Table 2). In addition, we failed to report any significant interaction between BMI and 25(OH)D in patients with NC (p = 0.57).

**Table 2:** Associations between 25-hydroxyvitamin D (25(OH)D) level and patients with narcolepsy-cataplexy.

	Normal controls N = 55		Narcolepsy with cataplexy N = 51					
	*n*	*%*	*n*	*%*	*OR [95%CI]*	*p-value* *	*OR [95%CI]* **	*p-value* **
**25(OH)D, nmol/L** 1								
<45.9	8	14.55	19	37.25	**5.34 [1.65**–**17.27]**	0.0023	**3.63 [1.06**–**12.46]**	0.0191
[45.9–70.4[	12	21.82	14	27.45	2.63 [0.84–8.17]		2.43 [0.76–7.77]	
[70.4–89.3[	17	30.91	10	19.61	1.32 [0.42–4.15]		1.19 [0.37–3.85]	
≥89.3	18	32.73	8	15.69	1		1	

1 Quartile of the whole population.

*For variables with more than two categories, the p-value of the test for trend is given.

**Adjustment for BMI on two classes (<25 and ≥25 kg/m^2^).

No correlation was found between 25(OH)D and age at onset, duration of disease, Epworth Sleepiness Scale, mean sleep latency, or number of SOREMPs on the MSLT in patients with NC. Finally, no differences were found in 25(OH)D level with cataplectic attack frequency or the presence of treatment intake i.e. stimulants and/or anticataplectic drugs.

## Discussion

This is the first report to investigate vitamin D status in patients with NC. Results show significantly lower 25(OH)D level in European patients with NC compared to age- and gender-matched controls. Vitamin D deficiency was strongly associated with narcolepsy by an odds ratio up to 5-fold, independently of socio-demographic and clinical characteristics.

Serum 25(OH)D is representative of the overall vitamin D stored in the body (D2+D3) [8]. 25(OH)D serum level differs according to gender, BMI, skin color, season of sampling, sun exposure duration, and food intake, and it declines with age [8]. The strength of the present analysis was in comparing vitamin D level in patients with NC to age-, gender-, and ethnicity-matched controls while taking into account two other potential confounding factors: BMI and season of blood sampling. We confirmed lower sera 25(OH)D level in obese subjects and in winter and fall seasons. The effect of season of blood sampling did not differ between patients and controls. In contrast, overweight and obesity were more frequent in NC. However, the association between 25(OH)D level and NC persisted after adjustment for BMI, and no between BMI and 25(OH)D interaction was found in NC.

Previous studies used cutoffs of 30–75 nmol/l for vitamin D, but experts currently recommend a minimum clinical cutoff of >75 nmol/l for optimal bone metabolism and other physiologic roles of vitamin D [8], [14]. However, this limit is not population-based, and depends mainly on sunshine exposure and less on food intake. Accordingly, vitamin D deficiency is frequently reported in normal controls, with an overlap between normal and pathological status. In the present study, although patients and healthy controls were all Caucasian and resided in southern France (a notably sunny area) we pinpointed a high frequency of vitamin D deficiency even in normal controls (half of controls were below this threshold, although they had significantly more vitamin D than NC patients).

How vitamin D impacts on the development of narcolepsy is still unclear. Recent evidence has highlighted many actions of vitamin D on the immune and central nervous system functions [15], [16]. Vitamin D modulates the activity of regulatory T lymphocyte cells, modifying the balance between the lymphocytes Th2 and Th1 [15]. Moreover, vitamin D was confirmed as a key environmental factor for autoimmune diseases, especially in the thoroughly investigated domain of MS [9]. As mentioned above, NC is considered to be an autoimmune disease leading to loss of hypocretin-containing neurons [1]. As suggested by low monozygotic twin concordance, environmental risk factors appear to be highly important in developing NC [7]. However, despite reports of streptococcal infections in patients with recent narcolepsy onset [17], little is known about the influence of environmental factors. As for NC, MS is closely associated with an HLA class II gene, namely HLA DRB1*1501, which is in tight linkage disequilibrium with DQB1*0602 in Caucasians. Recent studies support that vitamin D deficiency is a key factor in the causal cascade to MS [9], as the effects of vitamin D on HLA class II gene expression with a single vitamin D response element (VDRE) are specific to the promoter of the HLA-DRB1*15 haplotype in MS [18]. This biological gene–environment interaction needs to be further assessed in NC.

Numerous studies have reported that vitamin D plays a significant immunomodulatory role in autoimmune diseases [9], [14], [15]. However, the discovery of reduced serum vitamin D level in patients with NC needs careful analysis before vitamin D is acknowledged to play a part in its pathophysiology. For instance, it is arguable that the hypovitaminosis D reported in NC reflects less sun exposure due to daytime sleepiness. However, naps in NC are typically brief and therefore unlikely to cause significantly less time outdoors. Moreover, no vitamin D level difference was observed between patients regarding disease severity (daytime sleepiness and cataplexy), disease duration, or treatment intake. Finally, almost all the controls resided in the same area (Southern France) and were students and/or workers within the hospital being unlikely to get more sun exposure than the patients, who were more heterogeneous in terms of professional occupation.

The present cross-sectional study has some limitations. First, patients with NC were not HLA-matched to normal controls. Because the HLA DQB1*0602 allele is present in 20% of Caucasian controls, HLA-adjusted comparison would have required 250 control subjects, which would have been beyond the scope of this exploratory study. Second, sera were often taken after a lengthy disease duration, which may have decreased the chances of detecting hypovitaminosis D as a predictor of the disease. However, no correlation was found between 25(OH)D level, age at onset, and/or NC duration. Third, we were unable to obtain sun exposure data at the time of study, during their youth, or during gestation. Finally, this analysis did not control for dietary vitamin intake, which is nevertheless a relatively rare practice in France, especially in this age range.

In conclusion, we found for the first time that patients with NC show decreased serum vitamin D level compared to matched normal controls. Future larger sample studies should investigate the potential pathogenic role of hypovitaminosis D as a risk factor for NC and how it interacts in highly genetically predisposed patients with NC to ultimately trigger the disease. The next steps would be to study the potential vitamin D deficiency health-related consequences, and to focus on the utility of systematically assessing vitamin D status in patients with NC in order to correct potential deficiency. That could be of paramount importance close to disease onset in the aim of limiting the autoimmune process.
